# 

**Published:** 1895-07

**Authors:** 


### PERSONAL.

Dr. L. Pope, Jr., is conducting very active work in State association matters in New Hampshire, all of which tends to strengthen the profession in the Eastern States.

The warm friends of Dr. John W. Gadsden will be pained to learn that he is again seriously ill.

Prof. N. S. Mayo, who has been on the sick-list for some period of time, is again back to his duties.

Dr. Frank S. Billings, V.M., has decided to locate in Worcester, Mass., and will make a specialty of the treatment of barrenness in mares and cows.

Dr. James A. Waugh will be among those who intend taking a post-graduate course at the National Veterinary College.

Dr. J. P. Turner, veterinarian of the Sixth Cavalry, stationed at Fort Myer, Virginia, has been enjoying a month's leave of absence.

Dr. James B. Paige, Professor of Veterinary Science, Massachusetts Agricultural College, will spend a year abroad in pathological and bacteriological work among those well-known laboratories of research. We wish him every success in his prospective great opportunities.

Dr. John Robertson, veterinarian of U. S. Cavalry, is a candidate in the army ranks for a commissioned office.

### MARRIAGES AMONG VETERINARIANS.

On June 12, 1895, at the residence of the bride's parents, 1528 Diamond Street, Philadelphia, by the Rev. H. B. Garner, Dr. John P. Turner, of the Sixth United States Cavalry, Fort Myer, Virginia, and Miss Eva Beaumont, of Philadelphia.

On June 20, 1895, at Philadelphia, Dr. Louis A. Mansbach, of 863 North Sixth Street, Philadelphia, was united in the bonds of matrimony to Miss Cora Esslinger, of the same city.

On June 22, 1895, at Sardinia, Ohio, Dr. S. R. Howard, veterinary surgeon, of Hillsboro, Ohio, to Mrs. Irene Klintworth, of Sardinia.

